# Synthesis, Structure and Insecticidal Activities of Some Novel Amides Containing *N*-Pyridylpyrazole Moeities

**DOI:** 10.3390/molecules170910414

**Published:** 2012-08-31

**Authors:** Wei-Li Dong, Jing-Ying Xu, Li-Xia Xiong, Zheng-Ming Li

**Affiliations:** 1 Tianjin Key Laboratory on Technologies Enabling Development of Clinical Therapeutics and Diagnostics (Theranostics), School of Pharmacy, Tianjin Medical University, Tianjin 300070, China; 2 State Key Laboratory of Elemento-Organic Chemistry, National Pesticide Engineering Research Center, Nankai University, Tianjin 300071, China; Email: xujunying@mail.nankai.edu.cn (J.-Y.X.); xionglixia@nankai.edu.cn (L.-X.X.); zml@nankai.edu.cn (Z.-M.L.)

**Keywords:** amide, *N-*pyridylpyrazole, synthesis, crystal structure, insecticidal activity

## Abstract

In our search for environmentally benign insecticides with high activity, low toxicity and low residue, a novel series of amides containing *N-*pyridylpyrazole moieties were designed and synthesized. The structures of the title compounds were characterized and confirmed by ^1^H-NMR and elemental analysis. Furthermore, the structure of compound **7l** was determined by single crystal X-ray diffraction. The preliminary bioassay tests showed that some of them exhibited good insecticidal activities against *Mythimna separata* Walker, *Plutella xylostella* (Linnaeus, 1758) and *Laphygma exigua* Hübner.

## 1. Introduction

**Figure 1:**
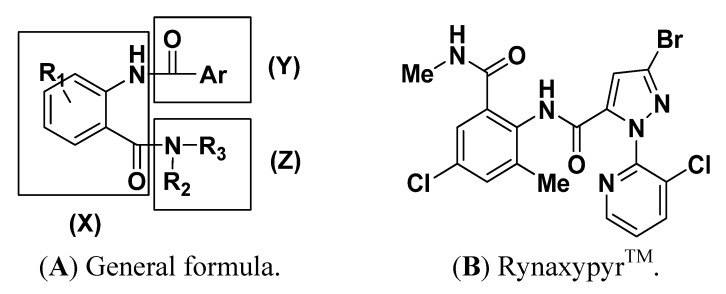
Chemical structures of anthranilic diamide insecticides.

Development of crop-protection molecules with unique modes of action is necessary to combat widespread insecticide resistance. Calcium channels, in particular, the ryanodine receptor (RyR) represent an attractive biological target for insect control and thus offers excellent promise in integrated pest management strategies [1]. Anthranilic diamides, discovered by DuPont, are a promising novel class of insecticides which exhibit their action by binding to insect ryanodine receptors (RyR) and activating the uncontrolled release of calcium stores [2,3,4]. Anthranilic diamide insecticides are characterized by a three-part chemical structure as shown in Figure 1A, where (**X**) is an anthraniloyl moiety, (**Y**) an aromatic acyl moiety and (**Z**) an aliphatic amide moiety. Notably, anthranilic diamides containing an *N-*pyridylpyrazole in the second section (**Y**) showed significantly better activity than other heterocyclic derivatives [5]. Work in this area has led to the discovery of Rynaxypyr^TM^ (Figure 1B), a highly potent and selective activator of insect ryanodine receptors with exceptional activity on a broad range of Lepidoptera, as the first new insecticide from this class [6].

In our previous work, when the *N-*pyridylpyrazole ring was replaced with 1,2,3-thiadiazole [7] or triazolopyrimidine [8] ones, the insecticidal activities were completely eliminated. In contrast, the modification of insecticidal anthranilic diamides with an ester group [9] or sulfonamide [10] substituting an amide group in the aliphatic amide moiety (**Z**) showed similar insecticidal activity, though of a lesser degree. Thus, these results suggest that the *N-*pyridylpyrazole unit plays an important role in the insecticidal activities of anthranilic diamides, but the aliphatic amide moiety (**Z**) may not be essential to insecticidal activities.

Encouraged by these reports, we developed an idea to examine whether the modification of the anthraniloyl skeleton by removing the aliphatic amide moiety (**Z**) could have an effect on potential insecticidal activities. Enlightened by all of the descriptions above, to further explore the comprehensive structure-activity relationships of the insecticidal activity, a series of novel amides containing *N*-pyridylpyrazoles were synthesized, and their insecticidal activities against *Mythimna separata* Walker, *Culex pipiens pallens*, *Plutella xylostella* (Linnaeus, 1758) and *Laphygma exigua* Hübner were tested and are discussed in this publication.

## 2. Results and Discussion

### 2.1. Chemistry

**Scheme 1:**
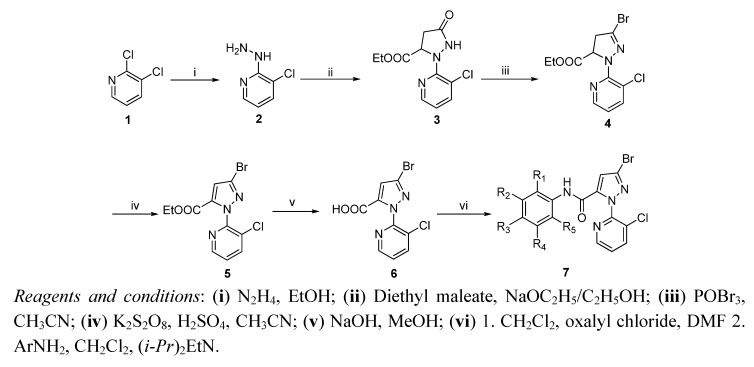
The synthetic route to title compounds **7**.

The synthetic route to the title compounds **7a**–**s** is shown in Scheme 1. The pyrazole carboxylic acid **6** is a key intermediate to the synthesis of target amides **7** containing *N*-pyridylpyrazoles. Various synthetic routes have been reported for the synthesis of intermediate pyrazole-5-carboxylic acid **6** [11,12]. Considering the practical application of the synthetic method, an alternate route for the preparation of pyrazole carboxylic acid **6** was developed. Reaction of 2,3-dichloropyridine (**1**) with hydrazine hydrate at reflux using ethanol as solvent gave 3-chloro-2-hydrazinylpyridine (**2**). Condensation of diethyl maleate with hydrazine **2** in the presence of sodium ethoxide afforded the pyrazolidinone **3**. Subsequent treatment of **3** with phosphorus oxybromide in acetonitrile afforded the pyrazoline **4**. A variety of reagents were explored for oxidation of **4** to the pyrazole **5**. We first chose potassium permanganate as oxidant, but pyrazole **5** was obtained in only 32% yield. Subsequently potassium persulfate was used to give **5** in a good yield. The intermediate **6** could be prepared by hydrolysis of **5** with known methods. Finally, pyrazole carboxylic acid **6** was treated with oxalyl chloride at reflux to give the corresponding acid chloride, which was then reacted with commercially available substituted anilined to afford the title compounds **7a**–**s**. The various amides **7a**–**s** containing *N*-pyridylpyrazoles prepared are listed in Table 1.

**Table 1:** List of *N*-pyridylpyrazole-containing amides **7a**–**s**.

Compd.	R_1_	R_2_	R_3_	R_4_	R_5_	Compd.	R_1_	R_2_	R_3_	R_4_	R_5_
**7a**	H	Cl	H	H	H	**7k **	CH_3_	H	H	H	NO_2_
**7b**	H	H	F	H	H	**7l**	Cl	H	NO_2_	H	H
**7c**	H	H	Cl	H	H	**7m**	Br	H	NO_2_	H	H
**7d**	H	H	I	H	H	**7n**	NO_2_	H	Cl	H	H
**7e**	H	H	NO_2_	H	H	**7o**	Cl	H	H	Cl	H
**7f**	H	H	OC_2_H_5_	H	H	**7p**	CH_3_	H	Cl	H	CH_3_
**7g**	H	Cl	F	H	H	**7q**	CH_3_	H	Br	H	CH_3_
**7h**	CH_3_	H	CH_3_	H	H	**7r**	CH_3_	H	NO_2_	H	Cl
**7i**	CH_3_	H	NO_2_	H	H	**7s**	CH_3_	H	Cl	H	NO_2_
**7j**	CH_3_	H	H	H	CH_3_						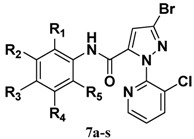

### 2.2. Crystal Structure

**Figure 2:**
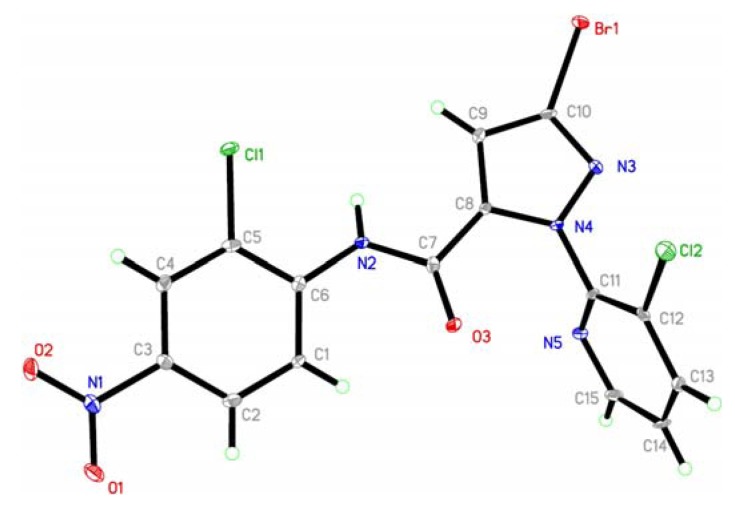
The molecular structure of **7l**.

**Figure 3:**
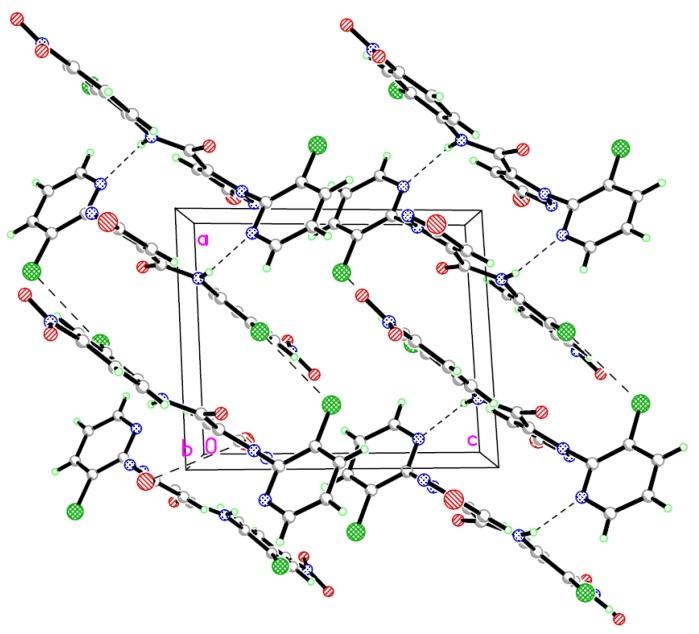
The packing of the molecules in the crystal lattice of **7l**.

The structure of compound **7l** was further confirmed by single crystal X-ray diffraction analysis (Figure 2 and Figure 3). In the molecular structure of title compound, the three ring (benzene ring, pyridine ring and pyrazole ring) are nearly vertically with θ angle of 80.6° (benzene ring *vs*. pyridine ring), 76.8° (pyrazole ring *vs*. pyridine) respectively, but the pyrazole ring is planar with the benzene ring (7.8°). 

The average bond lengths and bond angles of the phenyl ring [13,14,15,16,17], the pyrazole ring [18], pyridine ring [19] and the amide bond [20,21,22,23,24] are normal. The intermolecular edge-to-face π-π stacking appears between the pyridine ring and the phenyl ring in another adjacent molecule, in which the distance of H13 and the centroid of phenyl ring is 3.725 Å. These interactions can help to further stabilize the crystal structure. The title compound has an extensive network of hydrogen bonding involving the two acceptor N atoms. In the *bc* plane, they are linked together by N-H···N hydrogen bonds. This hydrogen-bonding sequence is repeated to form a ring.

### 2.3. Insecticidal Activities and Structure-Activity Relationship (SAR)

The insecticidal activities of all target compounds **7a**–**s** were determined *in vivo*. The results are summarized in Table 2. As shown, **7l** showed the most potent insecticidal activity against oriental armyworm (*M. separata*) in all the tested compounds, the death rate is 80% at 10 µg·mL^−1^. Compounds **7c**, **7i**, **7p**, **7q**, **7i** and **7r** also exhibited significant insecticidal activity against oriental armyworm, with death rates of more than 70% at 25 µg·mL^−1^.

**Table 2:** Insecticidal activity against *Mythimna separata* Walker and *Culex pipiens pallens* of title compounds (mortality/%).

Compd.	*Mythimna separata* Walker						*Culex pipiens pallens*
	µg·mL^−1^/death rate (%)						µg·mL^−1^/death rate (%)
	200	100	50	25	10	5	2
**7a**	0						— *^a^*
**7b**	100	100	100	50			0
**7c**	100	100	100	70	0		10
**7d**	100	100	50				100
**7e**	100	90	20				20
**7f**	100	0					30
**7g**	100	0					—
**7h**	100	70	0				—
**7i**	100	100	100	80	50		40
**7j**	100	80	40				—
**7k**	100	80	20				50
**7l**	100	100	100	100	80	0	80
**7m**	100	100	30				40
**7n**	10						40
**7o**	0						10
**7p**	100	100	100	80	20		30
**7q**	100	100	100	100	30		30
**7r**	100	100	100	70	30		—
**7s**	100	100	100	60			20
**Rynaxypyr^TM^**						100	100

*^a^* Not tested.

Further, the Structure-Activity Relationships (SAR) for different substitutions on the phenyl ring can be inferred from the results. Compound **7i** with an electron-donating methyl group at R^1^ exhibited similar insecticidal activity to that of compound **7l** with an electron-withdrawing chlorine group at R^1^, but the insecticidal activity decreased significantly when the chlorine atom was replaced with a bromine atom at R^1^ (compound **7m**). From these results, it appears that steric effects rather than electrostatic effects have substantial effects on the insecticidal activity at the R^1^. This observation appears consistent with the structure–activity of anthranilic diamides reported in the literature [25]. Among compounds **7b**–**e**, the electron-withdrawing group substituted analogues at R^3^ (compounds **7b**–**e**) were more active than the electron-donating group substituted analogue (compound **7f**). Besides, the electron-withdrawing halide substituted analogues at R^3^ (compounds **7p**, **7q**, **7s**) exhibited more insecticidal potency than unsubstituted analogs (compounds **7j**, **7k**). The electron-donating group substitution (compound **7j**) and electron-withdrawing group substitution (compound **7k**) showed similar levels of activity, and the presence of a chloro substituent at R^5^ (compound **7r**) and unsubstituted analog (compound **7i**) exhibited similar insecticidal activity. This result indicates that different substitutions at R^5^ did not exhibit significant influence on insecticidal activities. In addition, the insecticidal activity decreased significantly when electron-withdrawing chloro substituent was present at R^2^ and R^4^, such as **7a**, **7g**, **7o**. As shown in Table 2, the title compounds displayed good larvicidal activities against *C p. pallens*, for example, the larvicidal activity of **7d** was 100% at 2 µg·mL^−1^, as compared with 100% mortality of Rynaxypyr^TM^ at the same concentration, but showed no clear SAR trends.

**Figure 4:**
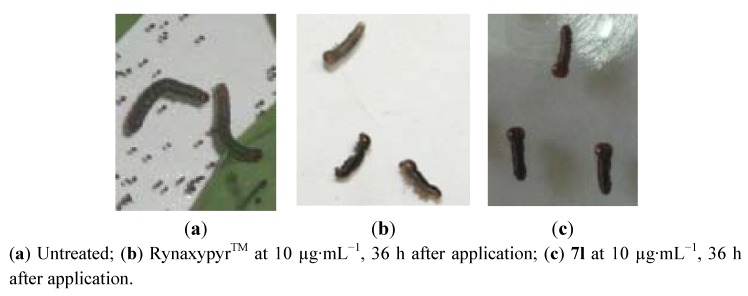
Symptoms of forth-instar larvae of *Mythimna separata* Walker treated by leaf dipping.

Figure 4 shows the symptoms of larvae affected by the title compounds and commercial Rynaxypyr^TM^. Insects treated with the title compound **7l** showed abnormal symptoms such as body contraction, vomiting, feeding cessation, body thickening and shortening, which are similar to those observed for larvae treated with commercial Rynaxypyr^TM^. These results suggest that the title compounds exhibit their activity by activating insect RyR.

For the compounds **7b**, **7c**, **7p**, **7q**, **7s**, **7l**, **7m**, further bioassay was conducted against *P. xylostella* and *L. exigua*. The results are summarized in Table 3. At a dose of 25 µg·mL^−1^, most of compounds have good insecticidal activity against *P. xylostella* and *L. exigua*, which can be compared with that of the control Rynaxypyr^TM^. This result showed that the title compounds have insecticidal activity on a broad spectrum of Lepidoptera.

**Table 3:** Insecticidal activity against *Plutella xylostella* (Linnaeus, 1758) and *Laphygma exigua* Hübner of title compounds (mortality/%).

Compd.	*Plutella xylostella* (Linnaeus, 1758)				*Laphygma exigua* Hübner			
	µg·mL^−1^/death rate (%)				µg·mL^−1^/death rate (%)			
	200	100	50	25	200	100	50	25
**7b**	100	98	99	88	95	100	92	96
**7c**	100	98	100	97	100	100	100	100
**7m**	100	98	96	90	100	100	100	100
**7l**	78	72	0		91	86	78	65
**7p**	100	100	97	94	100	100	98	94
**7q**	89	72	0	— *^a^*	100	100	89	93
**7s**	95	89	84	76	98	100	95	92
**Rynaxypyr** **^TM^**	—	—	100	94	—	—	100	100
**Ck**	0	0	0	0	0	0	0	0

*^a^* Not tested.

## 3. Experimental

### 3.1. General

Melting points were determined on an X-4 binocular microscope melting point apparatus and were uncorrected. ^1^H-NMR spectra were obtained on a Bruker AC-P500 spectrometer or Bruker Avance 400 spectrometer using tetramethylsilane (TMS) as an internal standard and CDCl_3_ or DMSO-*d_6_* as solvents. Elemental analyses were performed on a Vario EL elemental analyzer. High-resolution mass (HRMS) were recorded on a 7.0-T (Ionspec, Irvine, CA, USA) Fourier transform ion cyclotron resonance mass spectrometer. Crystallographic data of the compound **7l** were collected on a Rigaku Saturn diffractometer. All chemicals or reagents were purchased from standard commercial suppliers. Petroleum ether refers to the fraction of bp 60–90 °C.

### 3.2. Chemical Synthesis

#### 3.2.1. 3-Chloro-2-hydrazinylpyridine (**2**)

50% Hydrazine hydrate (200 mL).was added to a suspension of 2,3-dichloropyridine (**1**, 73.5 g, 0.5 mol) in anhydrous ethanol (300 mL). The resulting mixture was refluxed for 36 h, and then cooled to room temperature. A white crystalline product precipitated out of solution, which was collected by filtration, washed thoroughly with cold ethanol and dried to give hydrazine **2** (49.6 g, 69.0%), m.p. 163–164 °C; ^1^H-NMR (CDCl_3_, 400 MHz) *δ*: 3.97 (br s, 2H, NH_2_), 6.21 (br s, 1H, NH), 6.64 (m, 1H, pyridyl-H), 7.47 (d, *J* = 7.6 Hz, 1H, pyridyl-H), 8.09 (d, *J* = 4.9 Hz, 1H, pyridyl-H).

#### 3.2.2. Ethyl 2-(3-Chloro-2-pyridinyl)-5-oxopyrazolidine-3-carboxylate (**3**)

To absolute ethanol (200 mL) in a 500 mL three-necked round-bottomed flask was added sodium (6.9 g, 0.3 mol) cut into pieces of suitable size. After all the sodium had reacted, the mixture was heated to reflux and **2** (39.82 g, 0.277 mol) was added. The mixture was refluxed for 10 min, then diethyl maleate (51.65 g, 0.3 mol) was added dropwise. The resulting orange-red solution was held at reflux for 30 min. After being cooled to 65 °C, the reaction mixture was treated with glacial acetic acid (30 g, 0.51 mol). The mixture was diluted with water (30 mL). After removal of most of the solvent, the residue was treated with water (300 mL). The slurry formed was dissolved in aqueous ethanol (70%, 200 mL) and stirred thoroughly. The solid was collected by filtration, washed with aqueous ethanol (50%, 50 mL × 3) to give pyrazolidinone **3** (36.6 g, 49.0%), m.p. 132–134 °C; ^1^H-NMR (DMSO-*d_6_*, 300 MHz) *δ*: 1.20 (t, *J* = 6.8 Hz, 3H, CH_2_CH_3_), 4.18 (q, *J* = 7.2 Hz, 2H, CH_2_CH_3_), 2.34 (d, *J* = 16.8 Hz, 1H, CH_2_), 2.90 (q, *J* = 10.0 Hz, 1H, CH), 4.81 (d, *J* = 9.2 Hz, 1H, CH_2_), 7.18 (dd, *J* = 4.8, 7.6 Hz, 1H, pyridyl-H), 7.92 (d, *J* = 7.6 Hz, 1H, pyridyl-H), 8.25 (d, *J* = 4.0 Hz, 1H, pyridyl-H), 10.18 (br s, 1H, NH).

#### 3.2.3. Ethyl 3-Bromo-1-(3-chloro-2-pyridinyl)-4,5-dihydro-1H-pyrazole-5-carboxylate (**4**)

To a solution of **3** (0.1 mol) in acetonitrile (300 mL) was added phosphorus oxybromide (0.12 mmol). The reaction mixture was refluxed for 5 h, then most of the solvent (ca. 250 mL) was removed by distillation. The concentrated reaction mixture was slowly poured into saturated aq. Na_2_CO_3_ (250 mL) and stirred vigorously for 30 min. The resulting mixture was extracted with CH_2_Cl_2_ (250 mL × 2), the organic extract was separated, dried, filtered, concentrated and purified by silica gel chromatography to afford intermediates **4**. Yield 93.0%, m.p. 59–60 °C; ^1^H-NMR (DMSO-*d_6_*, 400 MHz) δ: 1.12 (t, *J* = 7.0 Hz, 3H, CH_2_CH_3_), 3.24–3.31 (m, 1H, CH_2_), 3.54–3.61 (m, 1H, CH_2_), 4.08 (q, *J* = 7.0 Hz, 2H, CH_2_CH_3_), 5.14–5.19 (m, 1H, CH), 6.98 (dd, *J* = 4.8, 7.6 Hz, 1H, pyridyl-H), 7.83 (d, *J* = 7.7 Hz, 1H, pyridyl-H), 8.10 (d, *J* = 4.4 Hz, 1H, pyridyl-H).

#### 3.2.4. Ethyl 3-Bromo-1-(3-chloro-2-pyridinyl)-1H-pyrazole-5-carboxylate (**5**)

To a solution of **4** (51 mmol) in acetonitrile (250 mL) was added sulfuric acid (98%, 10 g, 102 mmol). After being stirred for several minutes, the reaction mixture was treated with K_2_S_2_O_8_ (21 g, 76.5 mmol) and refluxed for 4.5 h. After being cooled to 60 °C, the mixture was filtered, the filter cake was washed with acetonitrile (30 mL). The filtrate was concentrated to 100 mL, then added slowly to water (250 mL) under stirring. The solid was collected by filtration, washed with acetonitrile (25%, 30 mL × 3), water (30 mL), and then dried to give intermediates **5**. Yield 92.7%, m.p. 117–118 °C; ^1^H-NMR (CDCl_3_, 400 MHz) *δ*: 1.21 (t, *J* = 6.8 Hz, 3H, CH_2_CH_3_), 4.22 (q, *J* = 7.2 Hz, 2H, CH_2_CH_3_), 7.03 (s, 1H, pyrazolyl-H), 7.44 (dd, *J* = 4.8, 8.4 Hz,1H, pyridyl-H), 7.91 (dd, *J* = 1.4, 8.0 Hz, 1H, pyridyl-H), 8.51 (dd, *J* = 1.4, 4.7 Hz, 1H, pyridyl-H). 

#### 3.2.5. 3-Bromo-1-(3-chloro-2-pyridinyl)-1H-pyrazole-5-carboxylic acid (**6**)

A mixture of **5** (47.2 mmol), methanol (120 mL), H_2_O (60 mL) and NaOH (2.3 g, 56.6 mmol) was stirred at room temperature for 6 h, then concentrated *in vacuo* to about 80 mL. The concentrated mixture was diluted with H_2_O (150 mL), and washed with ethyl acetate (150 mL). The aqueous solution was acidified using concentrated hydrochloric acid to pH 1.5. The solid was collected by filtration, washed with water (30 mL), and then dried to give pyrazolecarboxylic acid **6**. Yield 89.3%, m.p. 197–200 °C; ^1^H-NMR (CDCl_3_, 300 MHz) *δ*: 7.10 (s, 1H, pyrazolyl-H), 7.48 (dd, *J* = 4.8, 8.1 Hz, 1H, pyridyl-H), 7.94 (dd, *J* = 1.4, 8.0 Hz, 1H, pyridyl-H), 8.52 (dd, *J* = 1.4, 4.7 Hz, 1H, pyridyl-H). 

#### 3.2.6. General Procedure for the Synthesis of Compounds **7a**–**s**

To a suspension of *N*-pyridylpyrazole acid **6** (1 mmol) in dichloromethane (20 mL) was added oxalyl chloride (3 mmol), followed by dimethylformamide (2 drops). The solution was stirred at room temperature. After 3 h the mixture was concentrated in vacuo to obtain the crude acid chloride. The crude acid chloride in dichloromethane (10 mL) was added slowly to a stirred solution of substituted aniline **1** (1.2 mmol) in dichloromethane (20 mL) in an ice bath. After 20 min, diisopropylethylamine (1 mmol) was added dropwise. The solution was warmed to room temperature and stirred for 12 h, then diluted with CH_2_Cl_2_ (20 mL), and washed with 1 mol·L^−1^ aq. HCl solution (20 mL), saturated aq. NaHCO_3_ (20 mL), and brine (20 mL). The organic extract was separated, dried, filtered, concentrated and purified by silica gel chromatography to afford the desired *N*-pyridylpyrazole-containing amides **7**.

*3-Bromo-N-(3-chlorophenyl)-1-(3-chloropyridin-2-yl)-1H-pyrazole-5-carboxamide* (**7a**). Yield: 70.7%. White solid, m.p. 154–156 °C; ^1^H-NMR (CDCl_3_, 400 MHz) *δ*: 6.87 (s, 1H, pyrazolyl-H), 7.10–7.14 (m, 3H, Ar-H), 7.44 (dd, *J* = 4.8, 8.0 Hz, 1H, pyridyl-H), 7.58–7.59 (m, 1H, Ar-H), 7.93 (dd, *J* = 1.6, 8.0 Hz, 1H, pyridyl-H), 8.26 (br. s, NH), 8.48 (dd, *J* = 1.6, 4.8 Hz, 1H, pyridyl-H); Elemental anal. (%), calcd. for C_15_H_9_BrCl_2_N_4_O: C, 43.72; H, 2.20; N, 13.60; found: C, 43.56; H, 2.55; N, 13.30.

*3-Bromo-1-(3-chloropyridin-2-yl)-N-(4-fluorophenyl)-1H-pyrazole-5-carboxamide* (**7b**). Yield: 86.4%. White solid, m.p. 197–198 °C; ^1^H-NMR (CDCl_3_, 400 MHz) *δ*: 6.84 (s, 1H, pyrazolyl-H), 6.93–6.97 (m, 2H, Ar-H), 7.34–7.37 (m, 2H, Ar-H), 7.41 (dd, *J* = 4.8, 8.0 Hz, 1H, pyridyl-H), 7.91 (dd, *J* = 1.6, 8.0 Hz, 1H, pyridyl-H), 8.43–8.45 (m, 2H, pyridyl-H, NH); Elemental anal. (%), calcd. for C_15_H_9_BrClFN_4_O: C, 45.54; H, 2.29; N, 14.16; found: C, 45.65; H, 2.58; N, 13.90.

*3-Bromo-N-(4-chlorophenyl)-1-(3-chloropyridin-2-yl)-1H-pyrazole-5-carboxamide* (**7c**). Yield: 79.9%. White solid, m.p. 179–180 °C; ^1^H-NMR (CDCl_3_, 400 MHz) *δ*: 6.87 (s, 1H, pyrazolyl-H), 6.23–6.26 (m, 2H, Ar-H), 7.37–7.40 (m, 2H, Ar-H), 7.43 (dd, *J* = 4.8, 8.0 Hz, 1H, pyridyl-H), 7.93 (dd, *J* = 1.6, 8.0 Hz, 1H, pyridyl-H), 8.27 (br. s, NH), 8.47 (dd, 1H, *J* = 1.6, 4.8 Hz, pyridyl-H); Elemental anal. (%), calcd. for C_15_H_9_BrCl_2_N_4_O: C, 43.72; H, 2.20; N, 13.60; found: C, 43.82; H, 2.29; N, 13.53.

*3-Bromo-1-(3-chloropyridin-2-yl)-N-(4-iodophenyl)-1H-pyrazole-5-carboxamide* (**7d**). Yield: 62.3%. White solid, m.p. 198–201 °C; ^1^H-NMR (CDCl_3_, 400 MHz) *δ*: 6.87 (s, 1H, pyrazolyl-H), 7.21 (d, 2H, *J* = 8.8 Hz, Ar-H), 7.45 (dd, *J* = 4.8, 8.0 Hz, 1H, pyridyl-H), 7.58 (d, 2H, *J* = 8.4 Hz, Ar-H), 7.94 (dd, 1H, *J* = 1.6, 8.0 Hz, pyridyl-H), 8.275 (br. s, NH), 8.48 (dd, 1H, *J* = 1.6, 4.8 Hz, pyridyl-H); Elemental anal. (%), calcd. for C_15_H_9_BrClIN_4_O: C, 35.78; H, 1.80; N, 11.13; found: C, 36.14; H, 2.21; N, 10.82.

*3-Bromo-1-(3-chloropyridin-2-yl)-N-(4-nitrophenyl)-1H-pyrazole-5-carboxamide* (**7e**). Yield: 64.5%. White solid, m.p. 240–243 °C; 1H-NMR (CDCl3, 400 MHz) *δ*: 7.00 (s, 1H, pyrazolyl-H), 7.48 (dd, *J* = 4.8, 8.0 Hz, 1H, pyridyl-H), 7.69–7.71 (m, 2H, Ar-H), 7.96 (d, 1H, *J* = 8.0 Hz, pyridyl-H), 8.19–8.21 (m, 2H, Ar-H), 8.51 (d, 1H, *J* = 4.8 Hz, pyridyl-H), 8.70 (br. s, NH); Elemental anal. (%), calcd. for C_15_H_9_BrClN_5_O_3_: C, 42.63; H, 2.15; N, 16.57; found: C, 42.91; H, 2.48; N, 16.51.

*3-Bromo-1-(3-chloropyridin-2-yl)-N-(4-ethoxyphenyl)-1H-pyrazole-5-carboxamide* (**7f**). Yield: 90.9%. White solid, m.p. 191–193 °C; ^1^H-NMR (CDCl_3_, 400 MHz) *δ*: 1.39 (t, 3H, *J* = 6.8 Hz, CH_3_), 3.98 (q, 2H, *J* = 6.8 Hz, CH_2_), 6.79–6.81 (m, 2H, Ar-H), 6.84 (s, 1H, pyrazolyl-H), 7.31–7.33 (m, 2H, Ar-H), 7.41 (dd, *J* = 4.4, 8.0 Hz, 1H, pyridyl-H), 7.91 (dd, *J* = 1.6, 8.0 Hz, 1H, pyridyl-H), 8.15 (br. s, NH), 8.46 (dd, 1H, *J* = 1.6, 4.8 Hz, pyridyl-H); Elemental anal. (%), calcd. for C_17_H_14_BrClN_4_O_2_: C, 48.42; H, 3.35; N, 13.29; found: C, 48.66; H, 3.20; N, 12.91.

*3-Bromo-N-(3-chloro-4-fluorophenyl)-1-(3-chloropyridin-2-yl)-1H-pyrazole-5-carboxamide* (**7g**). Yield: 95.3%. White solid, m.p. 167–169 °C; ^1^H-NMR (CDCl_3_, 400 MHz) *δ*: 6.85 (s, 1H, pyrazolyl-H), 7.02–7.07 (m, 2H, Ar-H), 7.20–7.23 (m, 2H, Ar-H), 7.45 (dd, *J* = 4.8, 7.6 Hz, 1H, pyridyl-H), 7.63 (dd, 1H, *J* = 1.6, 5.6 Hz, pyridyl-H), 7.95 (d, 1H, *J* = 8.0 Hz, pyridyl-H), 8.35 (br. s, NH); Elemental anal. (%), calcd. for C_15_H_8_BrCl_2_FN_4_O: C, 41.89; H, 1.87; N, 13.03; found: C, 41.63; H, 2.17; N, 12.74.

*3-Bromo-1-(3-chloropyridin-2-yl)-N-(2,4-dimethylphenyl)-1H-pyrazole-5-carboxamide* (**7h**). Yield: 69.1%. White solid, m.p. 168–170 °C; ^1^H-NMR (CDCl_3_, 400 MHz) *δ*: 2.20 (s, 3H, CH_3_), 2.27 (s, 3H, CH_3_), 6.85 (s, 1H, pyrazolyl-H), 6.96–7.00 (m, 2H, Ar-H), 7.39 (dd, 1H, *J* = 4.8, 8.0 Hz, pyridyl-H), 7.46 (d, 1H, *J* = 8.0 Hz, Ar-H), 7.66 (br. s, 1H, NH), 7.87 (dd, 1H, *J* = 1.2, 8.0 Hz, pyridyl-H), 8.46 (dd, 1H, *J* = 1.6, 4.8 Hz, pyridyl-H; Elemental anal. (%), calcd. for C_17_H_14_BrClN_4_O: C, 50.33; H, 3.48; N, 13.81; found: C, 50.63; H, 3.50; N, 13.75.

*3-Bromo-1-(3-chloropyridin-2-yl)-N-(2-methyl-4-nitrophenyl)-1H-pyrazole-5-carboxamide* (**7i**). Yield: 62.3%. Yellow solid, m.p. 185–187 °C; ^1^H-NMR (DMSO-*d_6_*, 400 MHz) *δ*: 2.35 (s, 3H, CH_3_), 7.49 (s, 1H, Het-H), 7.61–7.67 (m, 2H, Ar-H), 8.06 (dd, *J* = 4.2 Hz, *J* = 4.4 Hz, 1H, Ar-H), 8.17 (d, *J* = 3.8 Hz, 1H, Py-H), 8.22 (d, *J* = 4.8 Hz, 1H, Py-H), 8.53 (dd, *J* = 4.5 Hz, *J* = 1.5 Hz, 1H, Py-H), 10.57(s, 1H, NH); Elemental anal. (%), calcd. for C_16_H_11_BrClN_5_O_3_: C, 44.01; H, 2.54; N, 16.04; found: C, 44.23; H, 2.38; N, 15.89.

*3-Bromo-1-(3-chloropyridin-2-yl)-N-(2,6-dimethylphenyl)-1H-pyrazole-5-carboxamide* (**7j**). Yield: 49.5%. White solid, m.p. 216–219 °C; ^1^H-NMR (CDCl_3_, 400 MHz) *δ*: 2.21 (s, 6H, CH_3_), 6.93 (s, 1H, pyrazolyl-H), 7.05–7.13 (m, 3H, Ar-H), 7.36–7.39 (m, 1H, pyridyl-H, 1H, NH), 7.87 (d, 1H, *J* = 6.8 Hz, pyridyl-H), 8.46 (d, 1H, *J* = 1.6, 4.4 Hz, pyridyl-H); Elemental anal. (%), calcd. for C_17_H_14_BrClN_4_O: C, 50.33; H, 3.48; N, 13.81; found: C, 50.03; H, 3.48; N, 13.81.

*3-Bromo-1-(3-chloropyridin-2-yl)-N-(2-methyl-6-nitrophenyl)-1H-pyrazole-5-carboxamide* (**7k**). Yield: 64.3%. White solid, m.p. 143-145 °C; ^1^H-NMR (DMSO-*d6*, 400 MHz) *δ*: 2.27 (s, 3H, CH_3_), 7.39 (s, 1H, pyrazolyl-H), 7.45 (t, 1H, *J* = 7.6 Hz, Ar-H), 7.61 (dd, *J* = 4.8, 8.0 Hz, 1H, pyridyl-H), 7.65 (d, 1H, *J* = 7.6 Hz, Ar-H), 7.79 (d, 1H, *J* = 7.6 Hz, Ar-H), 8.18 (dd, *J* = 1.2, 8.0 Hz, 1H, pyridyl-H), 8.50 (dd, 1H, *J* = 1.2, 4.4 Hz, 1H, pyridyl-H), 10.63 (br. s, NH); Elemental anal. (%), calcd. for C_16_H_11_BrClN_5_O_3_: C, 44.01; H, 2.54; N, 16.04; found: C, 44.25; H, 2.69; N, 15.85.

*3-Bromo-N-(2-chloro-4-nitrophenyl)-1-(3-chloropyridin-2-yl)-1H-pyrazole-5-carboxamide* (**7l**). Yield: 52.7%. White solid, m.p. 176–177 °C; ^1^H-NMR (CDCl_3_, 400 MHz) *δ*: 6.99 (s, 1H, pyrazolyl-H), 7.48 (dd, *J* = 4.8, 8.4 Hz, 1H, pyridyl-H), 7.96 (dd, *J* = 1.6, 8.4 Hz, 1H, pyridyl-H), 8.13 (dd, 1H, *J* = 2.4, 9.2 Hz, Ar-H), 8.34 (d, 1H, *J* = 2.4 Hz, Ar-H), 8.51 (dd, 1H, *J* = 1.2, 4.4 Hz, pyridyl-H), 8.55 (d, 1H, *J* = 9.2 Hz, Ar-H), 8.62 (br. s, 1H, NH); Elemental anal. (%), calcd. for C_15_H_8_BrCl_2_N_5_O_3_: C, 39.42; H, 1.76; N, 15.32; found: C, 39.09; H, 2.01; N, 15.51.

*3-Bromo-N-(2-bromo-4-nitrophenyl)-1-(3-chloropyridin-2-yl)-1H-pyrazole-5-carboxamide* (**7m**). Yield: 46.0%. White solid, m.p. 176–178 °C; ^1^H-NMR (CDCl_3_, 400 MHz) *δ*: 6.99 (s, 1H, pyrazolyl-H), 7.48 (dd, *J* = 4.8, 8.4 Hz, 1H, pyridyl-H), 7.96 (dd, *J* = 1.6, 8.0 Hz, 1H, pyridyl-H), 8.16 (dd, 1H, *J* = 2.4, 9.2 Hz, Ar-H), 8.49–8.53 (m, 1H, pyridyl-H, 2H, Ar-H), 8.60 (br. s, 1H, NH); Elemental anal. (%), calcd. for C_15_H_8_Br_2_ClN_5_O_3_: C, 35.92; H, 1.61; N, 13.96; found: C, 36.24; H, 1.73; N, 14.08.

*3-Bromo-N-(2-nitro-4-chlorophenyl)-1-(3-chloropyridin-2-yl)-1H-pyrazole-5-carboxamide* (**7n**). Yield: 55.5%. White solid, m.p. 193–194 °C; ^1^H-NMR (CDCl_3_, 400 MHz) *δ*: 7.02 (s, 1H, pyrazolyl-H), 7.46 (dd, *J* = 4.8, 8.0 Hz, 1H, pyridyl-H), 7.57 (dd, 1H, *J* = 2.8, 9.2 Hz, Ar-H), 7.94 (dd, *J* = 1.2, 8.0 Hz, 1H, pyridyl-H), 8.27 (d, 1H, *J* = 2.4 Hz, Ar-H), 8.51 (dd, 1H, *J* = 1.6, 4.8 Hz, pyridyl-H), 8.66 (d, 1H, *J* = 9.2 Hz, Ar-H), 11.14 (br. s, 1H, NH); Elemental anal. (%), calcd. for C_15_H_8_BrCl_2_N_5_O_3_: C, 39.42; H, 1.76; N, 15.32; found: C, 39.76; H, 1.98; N, 15.80.

*3-Bromo-1-(3-chloropyridin-2-yl)-N-(2,5-dichlorophenyl)-1H-pyrazole-5-carboxamide* (**7o**). Yield: 74.0%. White solid, m.p. 190–192 °C; ^1^H-NMR (CDCl_3_, 400 MHz) *δ*: 6.94 (s, 1H, pyrazolyl-H), 7.06–7.08 (m, 1H, Ar-H), 7.31–7.36 (m, 1H, Ar-H), 7.45–7.48 (m, 1H, pyridyl-H), 7.94 (d, 1H, *J* = 8.0 Hz, pyridyl-H), 8.35 (s, 1H, NH), 8.51 (d, 1H, *J* = 4.4 Hz, pyridyl-H); Elemental anal. (%), calcd. for C_15_H_8_BrCl_3_N_4_O: C, 40.35; H, 1.81; N, 12.55; found: C, 40.28; H, 2.06; N, 12.32.

*3-Bromo-N-(4-chloro-2,6-dimethylphenyl)-1-(3-chloropyridin-2-yl)-1H-pyrazole-5-carboxamide* (**7p**). Yield: 52.3%. White solid, m.p. 225–228 °C; ^1^H-NMR (CDCl_3_, 400 MHz) *δ*: 2.14 (s, 6H, CH_3_), 6.89 (s, 1H, pyrazolyl-H), 7.04 (s, 2H, Ar-H), 7.38 (dd, *J* = 4.8, 8.0 Hz, 1H, pyridyl-H), 7.45 (br. s, 1H, NH), 7.87 (dd, 1H, *J* = 1.6, 8.0 Hz, pyridyl-H), 8.44 (dd, 1H, *J* = 1.6, 4.4 Hz, pyridyl-H); Elemental anal. (%), calcd. for C_17_H_13_BrCl_2_N_4_O: C, 46.39; H, 2.98; N, 12.73; found: C, 46.18; H, 3.28; N, 12.17.

*3-Bromo-N-(4-bromo-2,6-dimethylphenyl)-1-(3-chloropyridin-2-yl)-1H-pyrazole-5-carboxamide* (**7q**). Yield: 55.2%. White solid, m.p. 237–238 °C; ^1^H-NMR (CDCl_3_, 400 MHz) *δ*: 2.17 (s, 6H, CH_3_), 6.92 (s, 1H, pyrazolyl-H), 7.22 (s, 2H, Ar-H), 7.38 (dd, *J* = 4.8, 8.0 Hz, 1H, pyridyl-H), 7.41 (br. s, 1H, NH), 7.86 (dd, 1H, *J* = 1.6, 8.0 Hz, pyridyl-H), 8.45 (dd, 1H, *J* = 1.6, 4.8 Hz, pyridyl-H); Elemental anal. (%), calcd. for C_17_H_13_Br_2_ClN_4_O: C, 46.99; H, 3.55; N, 13.70; found: C, 46.78; H, 3.38; N, 13.78.

*3-Bromo-N-(2-chloro-6-methyl-4-nitrophenyl)-1-(3-chloropyridin-2-yl)-1H-pyrazole-5-carboxamide* (**7r**). Yield: 53.2%. Yellow solid, m.p. 107–109 °C; ^1^H-NMR (DMSO-*d_6_*, 400 MHz) *δ*: 2.30 (s, 3H, CH_3_), 7.45 (s, 1H, pyrazolyl-H), 7.62 (dd, *J* = 4.4, 8.0 Hz, 1H, pyridyl-H), 8.18–8.23 (m, 2H, pyridyl-H, Ar-H), 7.80–7.82 (m, 1H, Ar-H), 8.34 (d, 1H, *J* = 2.4 Hz, Ar-H), 8.51 (dd, 1H, *J* = 1.2, 4.4 Hz, pyridyl-H), 10.79 (br. s, NH); Elemental anal. (%), calcd. for C_16_H_10_BrCl_2_N_5_O_3_: C, 40.79; H, 2.14; N, 14.87; found: C, 40.58; H, 2.21; N, 14.89.

*3-Bromo-N-(4-chloro-2-methyl-6-nitrophenyl)-1-(3-chloropyridin-2-yl)-1H-pyrazole-5-carboxamide* (**7s**). Yield: 46.6%. White solid, m.p. 100–104 °C; ^1^H-NMR (DMSO-*d_6_*, 400 MHz) *δ*: 2.28 (s, 3H, CH_3_), 7.01 (s, 1H, pyrazolyl-H), 7.40 (dd, *J* = 4.8, 8.4 Hz, 1H, pyridyl-H), 7.51 (d, 1H, *J* = 2.4 Hz, Ar-H), 7.87–7.90 (m, 2H, pyridyl-H, Ar-H), 8.47 (dd, 1H, *J* = 1.6, 4.8 Hz, pyridyl-H), 9.05 (br. s, 1H, NH); HRMS (ESI) *m/z*: 491.9233 (Calcd for C_16_H_10_BrCl_2_N_5_O_3_ [M+Na]^+^: 491.9236).

### 3.3. Crystal Structure Determination

The prism-shaped single crystal of the title compound was obtained by recrystallization from EtOH. The crystal with dimensions of 0.20 mm × 0.16 mm × 0.12 mm was mounted on a rigaku saturn diffractometer with a graphite-monochromated MoKα radiation (λ = 0.71073Å) by using a Phi scan modes at 113(2) K in the range of 2.28 ≤ θ ≤ 25.02. The crystals are triclinic, space group P*-1* with *a* = 8.8252(18), *b* = 9.1389(18), *c* = 10.448(2) Å, *α* = 96.61(3), *β* = 91.95(3), *γ* = 99.49(3)°, *V* = 824.4(3) Å^3^, *Z* = 2, *F(000)* = 452, *D_c_* = 1.841g/cm^3^, *μ* = 0.285 cm^−1^. A total of 5604 reflections were collected, of which 2884 were independent (*R*_int_ = 0.0345) and 2197 were observed with I > 2σ(I). The calculations were performed with SHELXS-97 program [26] and the empirical absorption corrections were applied to all intensity data. The non-hydrogen atoms were refined anisotropically. The hydrogen atoms were determined with theoretical calculations and refined isotropically. The final full-matrix least squares refinement gave: 



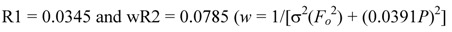



where *P* = (*F_o_*^2^ + 2*F_c_*^2^)/3, S = 1.07, (Δ/σ)_max_ = 0.002, Δρ_max_ = 0.46 and Δρ_min_ = −0.65 e Å^−3^.

Atomic scattering factors and anomalous dispersion corrections were taken from International Table for X-Ray Crystallography [27]. CCDC-893647 contains the supplementary crystallographic data for this paper. These data can be obtained free of charge at http://www.ccdc.cam.ac.uk/conts/retrieving.html or from the Cambridge Crystallographic Data Centre, 12 Union Road, Cambridge CB2 1EZ, UK; fax: +44-1223-336033; e-mail: deposit@ccdc.cam.ac.uk.

### 3.4. Biological Assay

All bioassays were performed on representative test organisms reared in the laboratory, which were repeated at 25 ± 1 °C according to statistical requirements. Assessments were made on a dead/alive basis and evaluations are based on a percentage scale of 0–100 in which 0 = no activity and 100 = total kill.

#### 3.4.1. Stomach Toxicity against *M. separata* Walker

The leaf dipping assay method was used for *M. separata* tests [28,29], in which the corn leaves were dipped into a test solution for 20 s and allowed to dry. The treated diet was placed into a 7 cm diameter Petri dish, and 10 fourth-instar *M. separata* larvae were released into the dish. The symptoms of affected larvae were observed at 24 h after the application, and percentage mortalities were evaluated 72 h after treatment. For comparative purposes, Rynaxypyr^TM^ was tested under the same conditions. Each treatment was performed three times.

#### 3.4.2. Toxicity against *Culex pipiens pallens*

The immersion method assay was used for *C. p. pallens* tests [30], and concentrations of test compounds were adjusted by serial dilution of a stock solution of the compounds in acetone. Each compound in acetone was suspended in distilled water, 10 early fourth-instar larvae of *C. p. pallens* were put into glass cups (125 mL) containing each test solution (100 mL). Larvicidal activity was evaluated 72 h after treatment. For comparative purposes, Rynaxypyr^TM^ was tested under the same conditions. Each treatment was performed three times.

#### 3.4.3. Stomach Toxicity against *Plutella xylostella* (Linnaeus, 1758) and *Laphygma exigua Hübner*

The leaf dipping assay method was used for *P. xylostella* and *L. exigua* tests [31,32]. A stock solution of each test sample was prepared in dimethylformamide at a concentration of 200 mg L^−1^ and then diluted to the required concentration with water containing TW-20. Leaf disks (6 cm × 2 cm) were cut from fresh cabbage leaves and then were dipped into the test solution for 3 s. After air-drying, the treated leaf disks were placed individually into glass tubes. Each dried treated leaf disk was infested with seven second-instar *P. xylostella* larvae (third-instar *L. exigua* larvae). Percentage mortalities were evaluated three days after treatment. Leaves treated with water and dimethylformamide were provided as controls. For comparative purposes, Rynaxypyr^TM^ was tested under the same conditions. Each treatment was performed three times.

## 4. Conclusions

In summary, a series of amides containing *N-*pyridylpyrazoles were synthesized and assessed for their insecticidal activities *in vivo*, using Rynaxypyr^TM^ as reference control. Several of the synthesized compounds exhibited significant insecticidal activity on a broad spectrum of Lepidoptera. Compared with the anthranilic diamide insecticide Rynaxypyr^TM^, the removal of the aliphatic amide moiety (**Z**) from the anthraniloyl skeleton resulted in slightly decreased insecticidal efficacy. This implies that the aliphatic amide moiety might not be the insecticidal pharmacophore. The present findings provided a powerful complement to the SARs of amide insecticides, and warrant future investigation of the mechanism of action of these analogues.
